# Prediction of Postoperative Mortality in Liver Transplantation in the Era of MELD-Based Liver Allocation: A Multivariate Analysis

**DOI:** 10.1371/journal.pone.0098782

**Published:** 2014-06-06

**Authors:** Helge Bruns, Vladimir J. Lozanovski, Daniel Schultze, Norbert Hillebrand, Ulf Hinz, Markus W. Büchler, Peter Schemmer

**Affiliations:** Department of General and Transplant Surgery, Ruprecht-Karls University, Heidelberg, Germany; The Chinese University of Hong Kong, Hong Kong

## Abstract

**Background and Aims:**

Liver transplantation is the only curative treatment for end-stage liver disease. While waiting list mortality can be predicted by the MELD-score, reliable scoring systems for the postoperative period do not exist. This study's objective was to identify risk factors that contribute to postoperative mortality.

**Methods:**

Between December 2006 and March 2011, 429 patients underwent liver transplantation in our department. Risk factors for postoperative mortality in 266 consecutive liver transplantations were identified using univariate and multivariate analyses. Patients who were <18 years, HU-listings, and split-, living related, combined or re-transplantations were excluded from the analysis. The correlation between number of risk factors and mortality was analyzed.

**Results:**

A labMELD ≥20, female sex, coronary heart disease, donor risk index >1.5 and donor Na^+^>145 mmol/L were identified to be independent predictive factors for postoperative mortality. With increasing number of these risk-factors, postoperative 90-day and 1-year mortality increased (0–1: 0 and 0%; 2: 2.9 and 17.4%; 3: 5.6 and 16.8%; 4: 22.2 and 33.3%; 5–6: 60.9 and 66.2%).

**Conclusions:**

In this analysis, a simple score was derived that adequately identified patients at risk after liver transplantation. Opening a discussion on the inclusion of these parameters in the process of organ allocation may be a worthwhile venture.

## Introduction

Liver transplantation is the only curative treatment for end-stage liver disease [1]. Today, this can be performed with low mortality rates and has become a standard technique since the 1980s [2]. Transplantation is a challenging surgical and medical field; pre- and postoperative logistics, infrastructural conditions in the transplant centers, experience of the surgeons involved, and anaesthesiological and medical - e.g. immunosuppressive – management strategies introduce complex factors that contribute to success of the procedure, but these factors might also influence postoperative mortality rates to varying degrees [3], [4].

Since the demand for organs is much higher than the number of available donor organs, patients on the waiting list for a liver transplant have to be prioritized. A MELD-based allocation system was introduced in December 2006 in the Eurotransplant region which was adopted from the UNOS region [5]. The MELD-based allocation strategy is sometimes described as a “sickest first” system since MELD-values are used to predict 3-month mortality probability of patients with end-stage liver disease who have not undergone liver transplantation, thus enabling surgeons to give priority to patients with higher scores [6]. The primary aim of MELD-based allocation – a reduction in the waiting list mortality rates – was accomplished in a relatively short timeframe [7], [8]. After the establishment of MELD-based allocation in the Eurotransplant region, median labMELD scores increased, indicating that “sicker” patients underwent liver transplantation first [8], [9]. Simultaneously, there was an increase in the postoperative mortality rates. High labMELD-scores were discussed as a possible risk factor for increased postoperative mortality [10], [11] and it has been demonstrated that, in comparision to other procedures, sicker patients who undergo liver transplantation not only consume more resources, but also have a higher probability for postoperative morbidity and mortality [6], [9]. High-MELD patients tend to have longer hospital stays and consume more blood-products [12].

Although an association between high MELD-scores and increased mortality has been reported in the literature [9], [11], no source has, to date, identified the factors that contribute to the correlation [10]. In the past few years, mortality and outcome after liver transplantation has continuously been investigated [2], [13]–[15]. It seems likely that there is a donor-specific influence on postoperative outcome [16], [17] therefore scores, such as D-MELD [18] and the donor risk index [19], have been derived from large retrospective analyses. Although these scores help to identify high risk patients, a precise method for predicting postoperative survival probability has not been accomplished as yet [20].

In general, transplantation aims to improve survivability and quality of life. It has clearly been shown that these two important parameters are improved by the inclusion of liver transplantation surgery in the patients' general medical care strategy [21]. Nonetheless, the postoperative mortality rate is still significant, therefore identifying the patients with increased risk is of great importance. Under German law, donor organs should be allocated according to the probability for “success” of the transplantation [22]. The associated ethical debate circles around the view that life is a universal value that is independent of its expected duration. The discussion on this subject must certainly include ways to identify the patients' postoperative survival probability, especially if patients who would benefit more from the best medical care management than from transplantation could be identified. While waiting list mortality rate can be predicted adequately using the MELD score [23], no accurate prediction for postoperative liver transplantation survival probabilities has been found [20]. Given that it is possible to identify a subgroup of patients who have a higher mortality probability after liver transplantation surgery, the current system of organ allocation might need to be refined.

Over the last few years, our center has become one of the largest German facilities for liver transplantation with highly standardized preoperative management strategies, surgical procedure and postoperative treatment [4], [24], and postoperative morbidity and mortality rates that are comparable to other large medical centers worldwide. This study aimed to identify risk factors associated with increased postoperative mortality in a typical patient collective undergoing liver transplantation and to establish a risk score that would help to identify high risk patients.

## Patients and Methods

### Patient collective and monitored parameters

**Figure 1:**
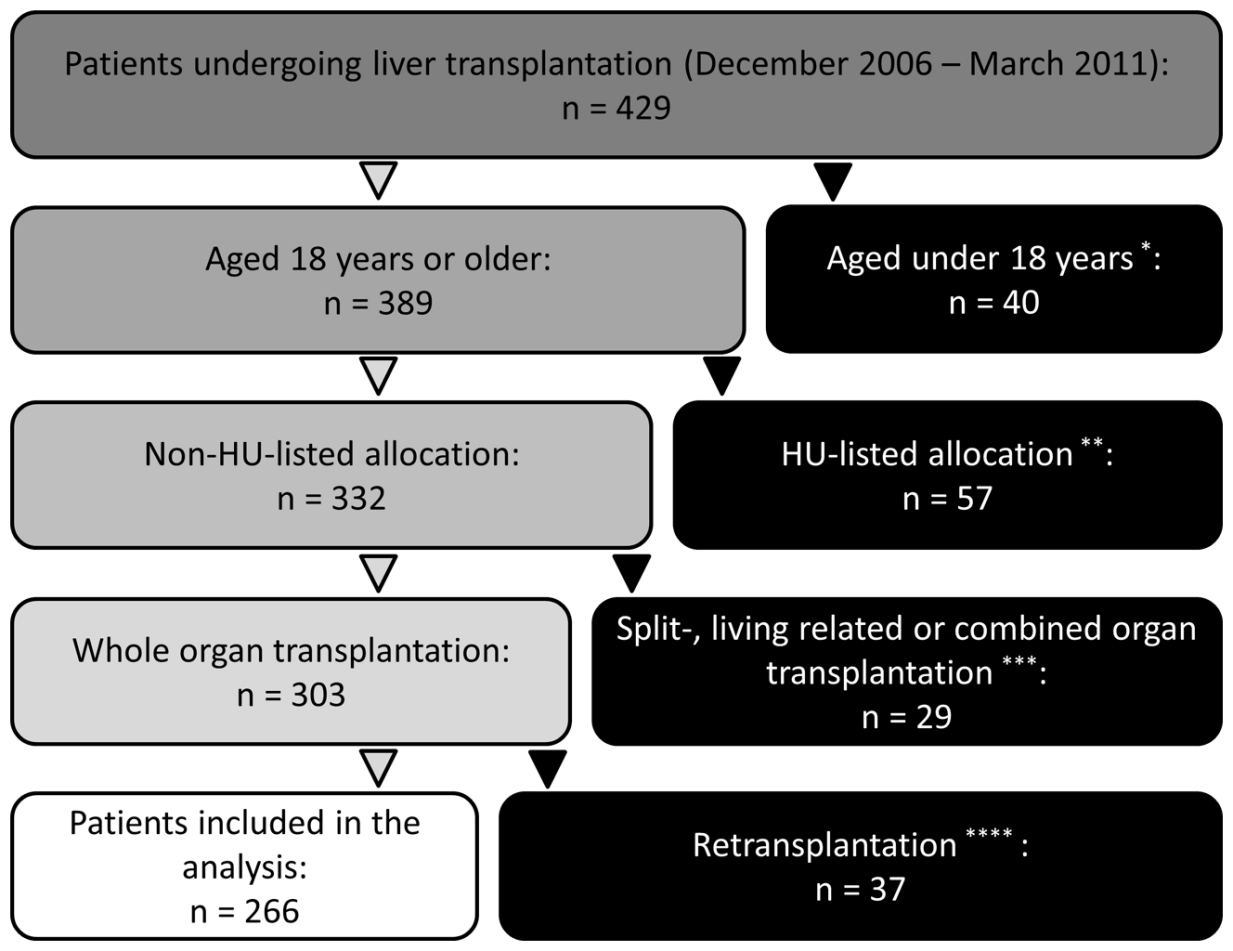
Patient Allocation. Of 429 patients who underwent liver transplantation at our institution from December 2006 till March 2011, 266 were eligible for inclusion in the study. ^*^incl. 18 HU-listed, 33 split/living related, and 5 retransplantations; excl. ^**^<18 years; ^***^<18 years or HU-listed; ^****^<18 years, HU-listed or split/living related or combined organ transplantation.

All national and institutional guidelines and regulations concerning data acquisition in retrospective analyses were followed at all times. After obtaining approval from the local ethics committee (Ethikkommission der Medizinischen Fakultät Heidelberg, Heidelberg, Germany; reference number: S-548/2012), the study was conducted with all patients who underwent liver transplantation at the University of Heidelberg from the establishment of MELD-based allocation in December 2006 until March 2011. According to the approval statement by the local ethics committee, no consent was needed for this single-center analysis. Only persons who were at least 18 years of age were included in the study. High urgency listed patients and persons who underwent living donor, split liver, combined or re-transplantations were excluded from the analysis. Of the 429 patients who had undergone liver transplantation procedures, 266 were eligible for inclusion in the study (Figure 1).

As a standard, pre-, peri-, and postoperative parameters concerning donor and recipient characteristics were monitored and collected in a database, and all data on patient survival probability rates was included in regular follow-up sessions. Complete datasets, including survival probability data, were available for all of the eligible patients for at least one year after surgery. The parameters under investigation are listed in Tables 1–3.

**Table 1:** Recipient characteristics.

	n	%
Patients included into the analysis	266	100
Survival		
<90 days	32	12
>90 days	234	88
	median	IQR
Age [years]	54	47–60
Sex	n	%
male	193	73
female	73	27
Indication		
cirrhosis (alcoholic)	81	30.45
cirrhosis (viral)	67	25.19
HCC	39	14.66
other	79	29.70
Comorbidities		
coronary heart disease	32	12.03
renal insufficiency	106	39.85
diabetes mellitus	65	24.44
encephalopathy	116	43.61
	Median	IQR
Body mass index	25.68	23.67–29.26
labMELD	16	10.25–29
NaMELD	20.1	11.91–30.74

**Table 2:** Donor characteristics.

	Median	IQR
Age	61	48–71
Sex	n	%
male	145	55
female	121	45
	Median	IQR
Donor risk index	1.87	1.52–2.06
Body mass index	26.04	23.46–27.78
Serum parameters	Mean	SD
Na^+^ [mmol/L]	148.37	10.29
CRP [mg/L]	141.82	110.91
creatinine [mg/dL]	1.43	2.78
AST [U/L]	80.20	94.60
ALT [U/L]	63.20	85.17
bilirubin [mg/dL]	0.67	0.57

**Table 3:** Perioperative parameters included in the analysis.

	Median	IQR
duration of surgery [hh:min]	05:30	04:30–06:35
suction bag volume* [ml]	3100	1500–6000
cold ischemic time [hh:min]	11:20	9:54–13:17
Transplant histology		
steatosis [%]	10	0–25
	n	%
Fibrosis	49	18.42

^*^total volume collected during surgery including ascites, rinsing fluids, and blood.

### Calculation of lab-, Na-, and D-MELD scores

labMELD scores were calculated according to the Mayo-Model (MELD = 3.78[Ln serum bilirubin (mg/dL)]+11.2[Ln INR]+9.57[Ln serum creatinine (mg/dL)]+6.43 [25]. When patients underwent dialysis during the last week of treatment or if creatinine levels were greater than 4.0 mg/dL, creatinine value in the calculations was set to 4.0 mg/dL. All variables that were less than 1 were set to 1. NaMELD scores were calculated as labMELD+1.59 (135 – Na^+^) with the maximum and minimum cutoff values for Na^+^ being 135 and 120 mmol/L [26] respectively. D-MELD scores were calculated as a product of donor age and preoperative labMELD values [18].

### Statistics

For descriptive statistics of the monitored parameters, the median and interquartile ranges (IQR), as well as the mean and standard deviations (SD) of the values obtained were used. Due to the low number of events, no stratification of labMELD scores and DRI was performed to identify possible cut-off values for the uni- and multivariate analysis; thus, cut-off values had to be defined in advance. For labMELD scores, a value of 20 was defined as cut off value and for DRI, the 1^st^ quartile was calculated and used. Hypo- and hypernatremia was defined as serum natrium below or above 135–145 mmol/L. Overall survival was defined as the elapsed time from the date of transplantation until death. The exact cause of death was irrelevant. Survival curves were constructed using the Kaplan-Meier estimator. A risk factor analysis was performed with respect to the 90-day mortality timeframe. The univariate analysis was performed using the logistic regression analysis and the Chi^2^-test or Fisher's exact test, where appropriate. Due to the low number of events, parameters with a *p* value of <0.10 were considered significant and included into the multivariate logistic regression analysis. In the final model of the multivariate regression analysis, only parameters with a *p* value of ≤0.05 or an odd ratio that did not include 1 were considered to be significant and parameters were described using odds ratio and 95% confidence interval values. Parameters that were included in the final model were used to construct a risk score. The patient collective was stratified into subgroups according to the number of positive risk factors. Differences between survival curves of patient subgroups were analyzed using the log-rank test. Unless otherwise stated, two sided *p* values were considered statistically significant at *p*<0.05.

## Results

Recipient and donor characteristics and intraoperative parameters are described in Tables 1–3. The univariate analysis identified eleven parameters (*recipient specific*: labMELD, NaMELD, sex, coronary heart disease, and renal insufficiency; *donor specific*: DRI, serum Na^+^, serum AST, and serum ALT; *surgery related*: intraoperatively collected suction bag volume and cold ischemia times) that had significant influence on the 90-day mortality rates after liver transplantation (Table 4). All of these factors were included in the multivariate analysis and five parameters (*recipient specific*: labMELD, coronary heart disease, sex; *donor specific*: DRI, serum Na^+^) that are preoperatively available had a significant influence on patient survival (Table 5).

**Table 4:** The Risk Factors identified in the univariate analysis.

Survival [days]	<90	≥90	*p*
Male recipient [%]	47	76	0.001
Coronary heart disease [%]	28	9.83	0.005
Renal insufficiency [%]	59.38	37.18	0.019
labMELD [median (IQR)]	28.5 (18.75–32.5)	15 (10–28)	0.001
NaMELD [median (IQR)]	29.39 (20.44–34.71)	18.79 (11.54–29.58)	0.005
suction bag volume* [ml; median (IQR)]	5000 (1950–8000)	3000 (1500–6000)	0.041
CIT [hh:min; median (IQR)]	12:29 (10:08–14:03)	11:18.5 (09:52.5–13:06)	0.041
DRI [median (IQR)]	1.92 (1.71–2.09)	1.84 (1.5–2.06)	0.069
Donor serum Na^+^ [mmol/L; mean ± SD]	134,97±5,79	148,19±10,45	^+^0.028/0.034
Donor serum AST [mmol/L; mean ± SD]	119,84±115,23	78,11±91,86	0.074
Donor serum ALT [mmol/L; mean ± SD]	63,50±62,34	62,31±84,39	0.040

CIT: cold ischemic time; DRI: donor risk index; hyper-/hyponatremia defined as Na^+^<135 and >145 mmol/L.

^*^total volume collected during surgery including ascites, rinsing fluids, and blood.

**Table 5:** Multivariate, logistic regression analysis of patient survival rates.

Variables	Category	OR	95% CI	p
labMELD	≥20 vs. <20	5.62	2.29–15.25	0.0003
Coronary heart disease	yes vs. no	4.12	1.39–12.17	0.0097
DRI	≥1.5 vs. <1.5	3.33	1.03–15.08	0.0705
Sex	female vs. male	3.17	1.34–7.59	0.0085
Donor Na^+^ [mmol/L]	>145 vs. ≤145	2.91	1.20–7.71	0.0228

Final model of the multivariate logistic regression analysis of clinical parameters associated with the 90-day timeframe for patient survival in the study population. Clinical parameters included in the multivariate analysis were significant at the 10% level in the univariate analysis. OR: odds ratio; CI: confidence interval; DRI: donor risk index.

Female patients had a higher median labMELD in comparison to male patients, but sex was later identified as an independent risk factor. Further analysis of the 90-day mortality rates for patients with labMELD scores of <20 and ≥20 revealed an increase in the mortality rate of female recipients with labMELD scores of ≥20 in comparison to male recipients (32.6 vs. 13.7%; *p*<0.05). There were no observable differences in values for patients with labMELD scores <20 (*female vs. male 90-day mortality*: 10 vs. 4.2%; *p*>0.05).

**Figure 2:**
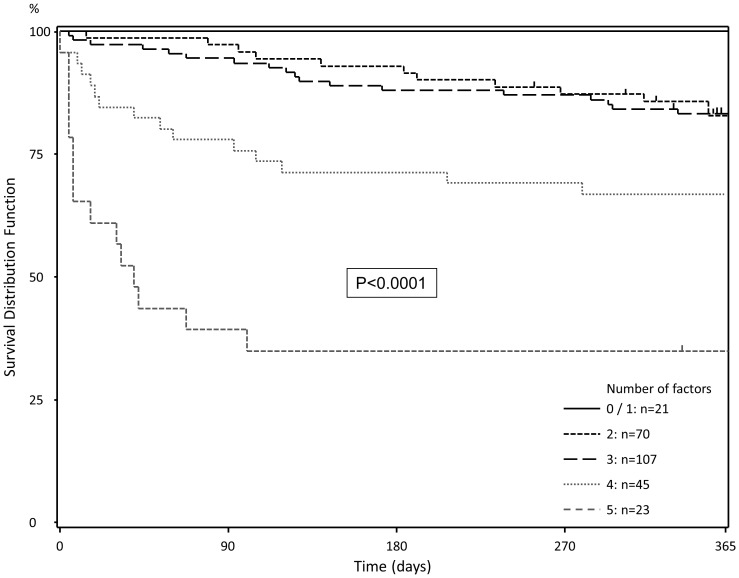
Survival stratified for number of positive risk factors. With an increasing number of risk-factors, postoperative 90-day and 1-year mortality increased (0–1: 0 and 0%; 2: 2.9 and 17.4%; 3: 5.6 and 16.8%; 4: 22.2 and 33.3%; 5: 60.9 and 66.2%).

**Figure 3:**
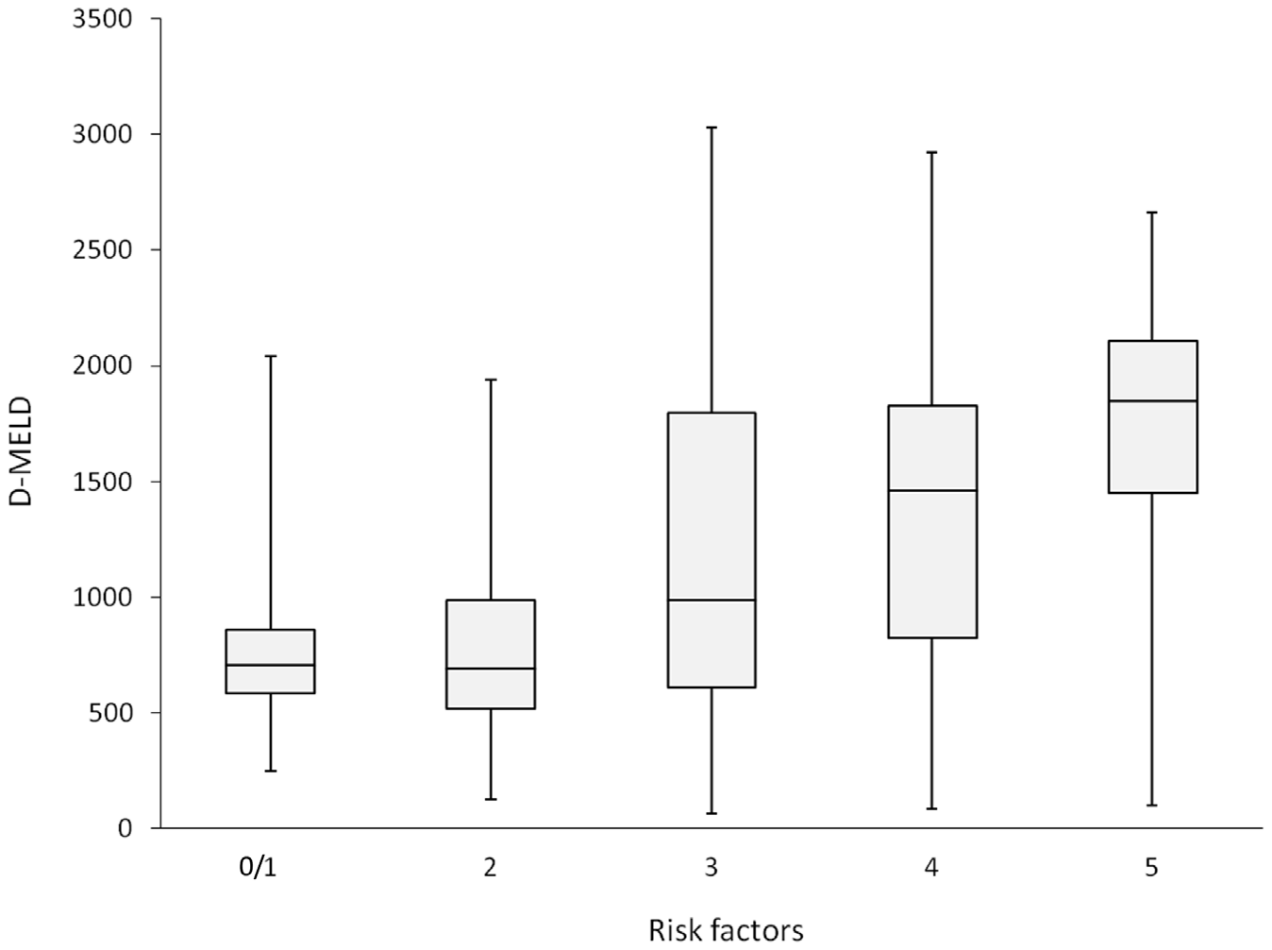
Box-Whisker-Plot depicting D-MELD scores for the number of positive risk factors in the patient collective. While there is an increase of median D-MELD-scores with an increasing number of risk factors, this is no significant correlation.

These factors were included in the risk score and patients were divided into subgroups according to the number of positive risk factors (Table 6, Figure 2). As the number of positive risk factors increases, there is an exponential hike in the 90-day mortality rates. Median D-MELD scores were 948.5 (IQR: 585.75–1673.5). Patients from both low (<1600) and high (≥1600) D-MELD groups had a median of 3 risk factors. While there was hike in the median D-MELD scores with an increase in the number of risk factors, no significant correlation could be detected between these two factors. Figure 3 illustrates this association between risk score and D-MELD. Moreover, while both the uni- and multivariate analyses indicated that the labMELD scores bore a significant influence, donor age was shown to have no impact on postoperative 90-day survival rates.

**Table 6:** Risk Factor score and patient survival rates.

		Survival [%]	
Number of risk factors	Number of patients	<90 days	≥365 days
0–1	21	100.00	100.00
2	70	97.10	82.60
3	107	94.40	83.20
4	45	77.78	66.70
5	23	39.13	34.80

## Discussion

In our patient collective, a clear correlation between the stated risk factors (Table 5) and postoperative mortality could be demonstrated (Table 6, Figure 2). With the exception of the warm ischemic time, all parameters are readily available before the actual transplantation operation takes place and hence could be included in a refined organ allocation system. In part, these parameters have been already identified as risk factors, since preoperative labMELD scores have been recognized as predictive parameters for patient postoperative survival rate, although no clear correlation between the two was previously identified [9]–[12].

When MELD-based allocation was established in Germany, an increase in the median labMELD scores of patients undergoing liver transplantation [6], [8], [9] and a simultaneous spike in patient postoperative mortality rates was observed. A significant correlation between the preoperative labMELD scores and postoperative mortality exists [6], [9]. This has been attributed to the fact that patients with higher MELD-scores are “sicker” and as such, have an increased risk of waiting list and intraoperative mortality. It can be stated that this risk persists after the early postoperative phase and in conjunction with comorbidties such as coronary heart disease, renal insufficiency, and diabetes mellitus, heightens the chances of waiting list mortality and a negative outcome after transplantation [27]–[29].

The parameters that predict postoperative survival are necessarily different from paramters that predict waiting list mortality. The underlying liver disease, patient age, immunosuppressive regimen, DRI and ischemic times have been identified as parameters and combinations with the MELD score have been discussed [30]–[33].

The amount of blood coagulation in a patient, which is determined by the International Normalized Ratio (INR), also has a significant impact on the patients' MELD scores. Patients undergoing liver transplantation with high MELD-scores are prone to blood loss and ultimately use more blood products than patients with low MELD scores. In our analysis, the total volume collected in the suction bag during surgery was used as a surrogate parameter to both blood loss and ascites. Although this was not identified as a significant parameter in the multivariate analysis, blood loss might be associated with reperfusion injury and persistent damage of the transplanted liver [34].

Coronary heart disease, a well-known risk factor for multiple types of surgery, has frequently been discussed as a risk factor for liver transplantation [35]. In contrast to the Hepatorenal Syndrome and renal insufficiency [36], it is not fully clear how coronary heart disease is linked to end-stage liver disease [37] but these patients clearly have an increased risk of mortality [38] independently of liver transplantation. While some literature sources have suggested that a protective effect of cirrhosis on atherosclerosis and myocardial infarction might exist [39], [40], there is a prevalence of coronary heart disease of up to 27% in patients with end-stage liver disease [35], [41]–[43]. In a retrospective analysis, a significant increase in the 1-year mortality rate was identified in patients with coronary heart disease after liver transplantation [35]. Coronary heart disease might be associated with hemodynamic and vascular alterations that typically contribute to an increased intraoperative mortality risk and also aggravate reperfusion injury, thus influencing postoperative survival rates via multiple pathways [44]–[46].

In several studies, ischemia/reperfusion injury has been identified as a relevant factor that leads to persistent postoperative liver damage [47], [48]. Both warm [49] and cold ischemic times seem to be relevant parameters that lead to inflammatory processes after reperfusion and contribute to persistent biliary complications [50], primary non function (PNF), and a delayed graft function [51], thereby decreasing the chances of a positive outcome after liver transplantation [52]. Although it has been suggested that setting a limit of 14–16 hours for cold storage in donors of up to 60 years old [47] and of 8–12 hours in donors over 60 years and with hepatosteatosis [53] prevents extensive cold ischemic damage to the liver, no limit has been suggested for the warm ischemic time since this value depends on the surgeons experience and is related to the anatomy of both the patient and the liver. The presence of other factors, such as steastosis [9], [54], before explantation [19] greatly influence reperfusion injury, primary dys- and non-function. This is reflected in the donor risk index [19]. This index has been shown to be effective in predicting postoperative liver function and survival rates in low-MELD patients, but not in high MELD patients [30], [31].

Our research indicated that female recipients were prone to postoperative mortality. Although an association between female donors and negative postoperative outcome has been shown in some studies [55]–[58], we did not identify this correlation in our patient collective. Brooks, *et al*. reported a decreased chance of survival when female livers were transplanted into male recipients [55]. Animal studies have revealed that estrogens have a biological influence on the long term postoperative outcome [59]. In contrast to these findings, our results indicated that it is the recipient's sex and not the donor's that had a significant influence on postoperative 90-day mortality rates. In general, female patients had higher labMELD scores when compared to male patients, but this did not explain the correlation that was observed in the multivariate analysis: when patients with labMELD scores of <20 and also ≥20 were categorized according to gender and 90-day mortality values, female recipients with high labMELD scores still had an increased mortality rate in comparison to their male counterparts (32.6 vs. 13.7%; *p*<0.05). In low labMELD patients, no significant difference between male and female recipients was seen (10 vs. 4.2%; *p*>0.05). With regard to all other monitored parameters, further analysis of the data did not identify any differences between male and female recipients.

In our study, Hypernatremia was identified as one of the six relevant risk factors for patient mortality. Donor Na^+^ has been discussed for quite some time as important influence on postoperative survival rates [60], [61]. High donor sodium levels may be due to an electrolyte and/or fluid imbalance which leads to an accumulation of intrahepatocellular osmolytes. The osmolytes protect against osmotic damage to the hepatocytes in the donor and, when the osmotic gradient increases in a normo- or hyponatremic recipient, this gives rise to increased intracellular pressure after reperfusion and subsequently results in cellular and liver damage [62]. The cellular release of transaminases in the recipient, which is a common indicator of liver damage, often corresponds to heightened donor sodium levels [60]–[62]. Both, the damage during reperfusion and recipient-specific factors that are not clearly related to reperfusion injury have a persistent effect on 90-day survival rates in patients.

In some publications, the D-MELD score, which is a product from the donor's age and preoperative labMELD score, is good identifying high risk patients [18]. As a rule of thumb, a D-MELD of 1600 is considered to be upper limit; any higher and postoperative mortality rates increase exponentially [18]. Although patients with high D-MELD scores (≥1600) had higher 90-day mortality rates, no significant correlation between D-MELD scores and the risk factor score was detected in our patient collective (Figure 3). Our multivariate analysis did not identify donor age to be a significant risk factor.

Postoperative mortality rates predicted by the risk score derived in this study should not be seen as fixed and the same may be true for the preoperative mortality rates predicted by the MELD score. With improved surgical technique, and better peri- and postoperative management, survival after liver transplantation has continuously improved over the decades and this trend will continue with the introduction of new immunosuppressive drugs and novel peri- and postoperative interventions [2]. In this context, this study does not aim to prevent transplantations of high risk patents, but rather to identify those who would benefit from individualized therapies and closer postoperative monitoring procedures.

Using our model, a female patient with a preoperative labMELD score of 20–30 (corresponding to a 3-month mortality of 11–49% without transplantation) and coronary heart disease should only get carefully selected organs. A DRI greater than 1.5 and donor Na^+^ levels higher than 145 mmol/L would, given that this score can be validated in a different patient collective, increase postoperative mortality to 60.1% - a rate much higher than that one predicted using only the MELD score without transplantation. But this is a constructed case and it is important to point out that the presence of these parameters do not necessarily mean that transplantation should not be performed. These values simply mean that postoperative procedures in these patients must be adapted and individualized in order to achieve a positive outcome. It might be noteworthy to discuss whether recipient specific parameters such as sex, comorbidities like coronary heart disease, DRI, and donor Na^+^ levels should be included in the allocation model, since these parameters are easily available during the process of organ allocation.

## Conclusions

This study clearly demonstrated that prediction of mortality risk after liver transplantation is possible. Five parameters (labMELD ≥20, female gender, coronary heart disease, donor risk index higher than 1.5, and donor Na^+^ levels >145 mmol/L) that are preoperatively available were identified to be independent predictive factors for postoperative 90-day and 1-year mortality. The ability to identify high risk patients after liver transplantation is a valuable asset that could lead to more effective, individualized postoperative therapies. Furthermore, it may be worthwhile to include predicted postoperative success rates in organ allocation strategies.

## References

[1] Merion RM (2010) Current status and future of liver transplantation. Semin Liver Dis 30: 411–421. 20960380 10.1055/s-0030-1267541

[2] Starzl TE, Klintmalm GB, Porter KA, Iwatsuki S, Schroter GP (1981) Liver transplantation with use of cyclosporin a and prednisone. N Engl J Med 305: 266–269. 7017414 10.1056/NEJM198107303050507 PMC2772056

[3] Jenkins RL, Fairchild RB (1989) The role of transplantation in liver disease. Surg Clin North Am 69: 371–382. 2648619 10.1016/s0039-6109(16)44792-4

[4] Schmied BM, Mehrabi A, Schallert C, Schemmer P, Sauer P, et al (2005) Evolution of liver transplantation at the University of Heidelberg: interventions influencing patient referral. Transplantation 80: S147–150. 16286894 10.1097/01.tp.0000186907.58294.d1

[5] Desschans B, Van Gelder F, Van Hees D, de Rocy J, Monbaliu D, et al (2008) Evolution in allocation rules for renal, hepatic, pancreatic and intestinal grafts. Acta Chir Belg 108: 31–34. 18411569

[6] Weismuller TJ, Fikatas P, Schmidt J, Barreiros AP, Otto G, et al (2011) Multicentric evaluation of model for end-stage liver disease-based allocation and survival after liver transplantation in Germany—limitations of the ‘sickest first’-concept. Transpl Int 24: 91–99. 20819196 10.1111/j.1432-2277.2010.01161.x

[7] Kim HJ, Larson JJ, Lim YS, Kim WR, Pedersen RA, et al (2010) Impact of MELD on waitlist outcome of retransplant candidates. Am J Transplant 10: 2652–2657. 21070603 10.1111/j.1600-6143.2010.03315.x PMC4547838

[8] Quante M, Benckert C, Thelen A, Jonas S (2012) Experience Since MELD Implementation: How Does the New System Deliver? Int J Hepatol 2012: 264015. 23091734 10.1155/2012/264015 PMC3467768

[9] Bruns H, Hillebrand N, Schneider T, Hinz U, Fischer L, et al (2011) LabMELD-based organ allocation increases total costs of liver transplantation: a single-center experience. Clin Transplant 25: E558–565. 21585550 10.1111/j.1399-0012.2011.01483.x

[10] Benckert C, Quante M, Thelen A, Bartels M, Laudi S, et al (2011) Impact of the MELD allocation after its implementation in liver transplantation. Scand J Gastroenterol 46: 941–948. 21443420 10.3109/00365521.2011.568521

[11] Tsui TY, Scherer MN, Schnitzbauer AA, Schlitt HJ, Obed A (2009) Adult living donor liver transplantation: body mass index and MELD score of recipients are independent risk factors for hospital mortality. Langenbecks Arch Surg 394: 235–241. 18512070 10.1007/s00423-008-0348-9

[12] David AI, Coelho MP, Paes AT, Leite AK, Della Guardia B, et al (2012) Liver transplant outcome: a comparison between high and low MELD score recipients. Einstein (Sao Paulo) 10: 57–61. 23045827 10.1590/s1679-45082012000100012

[13] Schmidt J, Muller SA, Mehrabi A, Schemmer P, Buchler MW (2008) Orthotopic liver transplantation. Techniques and results. Chirurg 79: 112–120. 18209990 10.1007/s00104-007-1452-z

[14] Venettoni S, Scalamogna M, Curtoni ES, Adorno D, Marino IR, et al (2004) Transplant quality in Italy: analysis of the 1995–2000 period. Transpl Int 17: 402–415. 15338115 10.1007/s00147-004-0718-3

[15] Weiss KH, Gotthardt D, Schmidt J, Schemmer P, Encke J, et al (2007) Liver transplantation for metabolic liver diseases in adults: indications and outcome. Nephrol Dial Transplant 22 Suppl 8viii9–viii12. 17890269 10.1093/ndt/gfm658

[16] Afonso RC, Hidalgo R, Paes AT, Zurstrassen MP, Fonseca LE, et al (2008) Impact of cumulative risk factors for expanded criteria donors on early survival after liver transplantation. Transplant Proc 40: 800–801. 18455021 10.1016/j.transproceed.2008.03.017

[17] Mateo R, Cho Y, Singh G, Stapfer M, Donovan J, et al (2006) Risk factors for graft survival after liver transplantation from donation after cardiac death donors: an analysis of OPTN/UNOS data. Am J Transplant 6: 791–796. 16539637 10.1111/j.1600-6143.2006.01243.x

[18] Halldorson JB, Bakthavatsalam R, Fix O, Reyes JD, Perkins JD (2009) D-MELD, a simple predictor of post liver transplant mortality for optimization of donor/recipient matching. Am J Transplant 9: 318–326. 19120079 10.1111/j.1600-6143.2008.02491.x

[19] Feng S, Goodrich NP, Bragg-Gresham JL, Dykstra DM, Punch JD, et al (2006) Characteristics associated with liver graft failure: the concept of a donor risk index. Am J Transplant 6: 783–790. 16539636 10.1111/j.1600-6143.2006.01242.x

[20] Schrem H, Reichert B, Fruhauf N, Becker T, Lehner F, et al (2012) The Donor-Risk-Index, ECD-Score and D-MELD-Score all fail to predict short-term outcome after liver transplantation with acceptable sensitivity and specificity. Ann Transplant 17: 5–13. 10.12659/aot.883452 23018250

[21] Togashi J, Sugawara Y, Akamatsu N, Tamura S, Yamashiki N, et al.. (2013) Quality of life after adult living donor liver transplantation: A longitudinal prospective follow-up study. Hepatol Res. 10.1111/hepr.12060 23369201

[22] Wheeler MD, Ikejema K, Enomoto N, Stacklewitz RF, Seabra V, et al (1999) Glycine: a new anti-inflammatory immunonutrient. Cell Mol Life Sci 56: 843–856. 11212343 10.1007/s000180050030 PMC11147092

[23] Wiesner R, Edwards E, Freeman R, Harper A, Kim R, et al (2003) Model for end-stage liver disease (MELD) and allocation of donor livers. Gastroenterology 124: 91–96. 12512033 10.1053/gast.2003.50016

[24] Mieth M, Schemmer P, Encke J, Weigand M, Weitz J, et al.. (2006) Heidelberger Manual der Lebertransplantation.

[25] Kamath PS, Wiesner RH, Malinchoc M, Kremers W, Therneau TM, et al (2001) A model to predict survival in patients with end-stage liver disease. Hepatology 33: 464–470. 11172350 10.1053/jhep.2001.22172

[26] Kim WR, Biggins SW, Kremers WK, Wiesner RH, Kamath PS, et al (2008) Hyponatremia and mortality among patients on the liver-transplant waiting list. N Engl J Med 359: 1018–1026. 18768945 10.1056/NEJMoa0801209 PMC4374557

[27] Fuller JH, Stevens LK, Wang SL (2001) Risk factors for cardiovascular mortality and morbidity: the WHO Mutinational Study of Vascular Disease in Diabetes. Diabetologia 44 Suppl 2S54–64. 11587051 10.1007/pl00002940

[28] Rossi E (1997) Cardiovascular disease in diabetes and operative risk. Rays 22: 595–602. 9550900

[29] Susantitaphong P, Altamimi S, Ashkar M, Balk EM, Stel VS, et al (2012) GFR at initiation of dialysis and mortality in CKD: a meta-analysis. Am J Kidney Dis 59: 829–840. 22465328 10.1053/j.ajkd.2012.01.015 PMC3395227

[30] Avolio AW, Siciliano M, Barbarino R, Nure E, Annicchiarico BE, et al (2008) Donor risk index and organ patient index as predictors of graft survival after liver transplantation. Transplant Proc 40: 1899–1902. 18675083 10.1016/j.transproceed.2008.05.070

[31] Bonney GK, Aldersley MA, Asthana S, Toogood GJ, Pollard SG, et al (2009) Donor risk index and MELD interactions in predicting long-term graft survival: a single-centre experience. Transplantation 87: 1858–1863. 19543065 10.1097/TP.0b013e3181a75b37

[32] Buenadicha AL, Martin LG, Martin EE, Pajares A, Perez AM, et al (2005) Assessment of short-term survival after liver transplant by the Model for End-Stage Liver Disease. Transplant Proc 37: 3881–3883. 16386571 10.1016/j.transproceed.2005.09.165

[33] Edwards E, Harper A (2004) Does MELD work for relisted candidates? Liver Transpl 10: S10–16. 15382287 10.1002/lt.20271

[34] Boin IF, Leonardi MI, Luzo AC, Cardoso AR, Caruy CA, et al (2008) Intraoperative massive transfusion decreases survival after liver transplantation. Transplant Proc 40: 789–791. 18455018 10.1016/j.transproceed.2008.02.058

[35] Yong CM, Sharma M, Ochoa V, Abnousi F, Roberts J, et al (2010) Multivessel coronary artery disease predicts mortality, length of stay, and pressor requirements after liver transplantation. Liver Transpl 16: 1242–1248. 21031539 10.1002/lt.22152

[36] Rahimi RS, Rockey DC (2013) End-stage liver disease complications. Curr Opin Gastroenterol. 10.1097/MOG.0b013e32835f43b0 23429468

[37] Garg A, Armstrong WF (2013) Echocardiography in liver transplant candidates. JACC Cardiovasc Imaging 6: 105–119. 23328568 10.1016/j.jcmg.2012.11.002

[38] Filippone EJ, Foy A (2012) The J-curve revisited: a therapeutic dilemma. Cardiol Rev 20: 253–258. 22447043 10.1097/CRD.0b013e3182564a34

[39] Carey WD, Dumot JA, Pimentel RR, Barnes DS, Hobbs RE, et al (1995) The prevalence of coronary artery disease in liver transplant candidates over age 50. Transplantation 59: 859–864. 7701580

[40] Turner TB, Bennett VL, Hernandez H (1981) The beneficial side of moderate alcohol use. Johns Hopkins Med J 148: 53–63. 7206400

[41] Donovan CL, Marcovitz PA, Punch JD, Bach DS, Brown KA, et al (1996) Two-dimensional and dobutamine stress echocardiography in the preoperative assessment of patients with end-stage liver disease prior to orthotopic liver transplantation. Transplantation 61: 1180–1188. 8610415 10.1097/00007890-199604270-00011

[42] Plotkin JS, Scott VL, Pinna A, Dobsch BP, De Wolf AM, et al (1996) Morbidity and mortality in patients with coronary artery disease undergoing orthotopic liver transplantation. Liver Transpl Surg 2: 426–430. 9346688 10.1002/lt.500020604

[43] Tiukinhoy-Laing SD, Rossi JS, Bayram M, De Luca L, Gafoor S, et al (2006) Cardiac hemodynamic and coronary angiographic characteristics of patients being evaluated for liver transplantation. Am J Cardiol 98: 178–181. 16828588 10.1016/j.amjcard.2006.01.089

[44] Carbo J, Garcia-Samaniego J, Castellano G, Iniguez A, Solis-Herruzo JA (1998) Liver cirrhosis and mortality by abdominal surgery. A study of risk factors. Rev Esp Enferm Dig 90: 105–112. 9580437

[45] Porte RJ (1993) Coagulation and fibrinolysis in orthotopic liver transplantation: current views and insights. Semin Thromb Hemost 19: 191–196. 8362248 10.1055/s-2007-994025

[46] Stahl RL, Duncan A, Hooks MA, Henderson JM, Millikan WJ, et al (1990) A hypercoagulable state follows orthotopic liver transplantation. Hepatology 12: 553–558. 2401460 10.1002/hep.1840120317

[47] Porte RJ, Ploeg RJ, Hansen B, van Bockel JH, Thorogood J, et al (1998) Long-term graft survival after liver transplantation in the UW era: late effects of cold ischemia and primary dysfunction. European Multicentre Study Group. Transpl Int 11 Suppl 1S164–167. 9664970 10.1007/s001470050452

[48] Zhai Y, Busuttil RW, Kupiec-Weglinski JW (2011) Liver ischemia and reperfusion injury: new insights into mechanisms of innate-adaptive immune-mediated tissue inflammation. Am J Transplant 11: 1563–1569. 21668640 10.1111/j.1600-6143.2011.03579.x PMC3658307

[49] Gastaca M (2012) Biliary complications after orthotopic liver transplantation: a review of incidence and risk factors. Transplant Proc 44: 1545–1549. 22841209 10.1016/j.transproceed.2012.05.008

[50] Scotte M, Dousset B, Calmus Y, Conti F, Houssin D, et al (1994) The influence of cold ischemia time on biliary complications following liver transplantation. J Hepatol 21: 340–346. 7836702 10.1016/s0168-8278(05)80311-3

[51] Piratvisuth T, Tredger JM, Hayllar KA, Williams R (1995) Contribution of true cold and rewarming ischemia times to factors determining outcome after orthotopic liver transplantation. Liver Transpl Surg 1: 296–301. 9346586 10.1002/lt.500010505

[52] Totsuka E, Fung JJ, Lee MC, Ishii T, Umehara M, et al (2002) Influence of cold ischemia time and graft transport distance on postoperative outcome in human liver transplantation. Surg Today 32: 792–799. 12203057 10.1007/s005950200152

[53] Yersiz H, Shaked A, Olthoff K, Imagawa D, Shackleton C, et al (1995) Correlation between donor age and the pattern of liver graft recovery after transplantation. Transplantation 60: 790–794. 7482736

[54] Bruns H, Watanpour I, Gebhard MM, Flechtenmacher C, Galli U, et al (2011) Glycine and taurine equally prevent fatty livers from Kupffer cell-dependent injury: an in vivo microscopy study. Microcirculation 18: 205–213. 21175929 10.1111/j.1549-8719.2010.00078.x

[55] Brooks BK, Levy MF, Jennings LW, Abbasoglu O, Vodapally M, et al (1996) Influence of donor and recipient gender on the outcome of liver transplantation. Transplantation 62: 1784–1787. 8990363 10.1097/00007890-199612270-00017

[56] Kahn D, Gavaler JS, Makowka L, van Thiel DH (1993) Gender of donor influences outcome after orthotopic liver transplantation in adults. Dig Dis Sci 38: 1485–1488. 8344105 10.1007/BF01308608

[57] Kahn D, Zeng QH, Makowka L, Murase N, Nakajima Y, et al (1989) Orthotopic liver transplantation and the cytosolic estrogen-androgen receptor status of the liver: the influence of the sex of the donor. Hepatology 10: 861–866. 2807167 10.1002/hep.1840100519 PMC2978925

[58] Marino IR, Doyle HR, Aldrighetti L, Doria C, McMichael J, et al (1995) Effect of donor age and sex on the outcome of liver transplantation. Hepatology 22: 1754–1762. 7489985 PMC2965620

[59] Jin B, Liu CZ, Hu SY, Wang TT, Wang L, et al (2008) Influence of estrogen and androgen on the outcome of liver transplantation. Hepatogastroenterology 55: 207–211. 18507108

[60] Avolio AW, Agnes S, Magalini SC, Foco M, Castagneto M (1991) Importance of donor blood chemistry data (AST, serum sodium) in predicting liver transplant outcome. Transplant Proc 23: 2451–2452. 1926428

[61] Kaseje N, Luthold S, Mentha G, Toso C, Belli D, et al (2013) Donor hypernatremia influences outcomes following pediatric liver transplantation. Eur J Pediatr Surg 23: 8–13. 23165516 10.1055/s-0032-1329703

[62] Cywinski JB, Mascha E, Miller C, Eghtesad B, Nakagawa S, et al (2008) Association between donor-recipient serum sodium differences and orthotopic liver transplant graft function. Liver Transpl 14: 59–65. 18161840 10.1002/lt.21305

